# Supplementary material for: Elevated SLC1A5 links to inflamed endothelial cells and proteinuria in membranous nephropathy patients
Source: PeerJ. 2025 Oct 31;13:e20271. doi: 10.7717/peerj.20271 (PMC12581921; doi:10.7717/peerj.20271)
Supplement: Supplemental Information 1 [file peerj-13-20271-s001.docx]

**STROBE-MR checklist of recommended items to address in reports of Mendelian randomization studies**^1^ ^2^

| **Item No.** | **Section** | **Checklist item** | **Page No.** | **Relevant text from manuscript** |
| --- | --- | --- | --- | --- |
| 1 | **TITLE and ABSTRACT** | Indicate Mendelian randomization (MR) as the study’s design in the title and/or the abstract if that is a main purpose of the study | 2 | hdWGCNA analysis identified three molecular sets linked to inflammatory cells, with Mendelian randomization confirming a causal relationship between these sets and MN onset. |
|  | **INTRODUCTION** |  |  |  |
| 2 | **Background** | Explain the scientific background and rationale for the reported study. What is the exposure? Is a potential causal relationship between exposure and outcome plausible? Justify why MR is a helpful method to address the study question | 4 | High-dimensional weighted gene co-expression network analysis (hdWGCNA) identified essential genes involved in the evolution of these cells, while MR established a link between SLC1A5 and MN pathogenesis. |
| 3 | **Objectives** | State specific objectives clearly, including pre-specified causal hypotheses (if any). State that MR is a method that, under specific assumptions, intends to estimate causal effects | 4 | High-dimensional weighted gene co-expression network analysis (hdWGCNA) identified essential genes involved in the evolution of these cells, while MR established a link between SLC1A5 and MN pathogenesis. |
|  | **METHODS** |  |  |  |
| 4 | **Study design and data sources** | Present key elements of the study design early in the article. Consider including a table listing sources of data for all phases of the study. For each data source contributing to the analysis, describe the following: |  |  |
|  | a) | Setting: Describe the study design and the underlying population, if possible. Describe the setting, locations, and relevant dates, including periods of recruitment, exposure, follow-up, and data collection, when available. | 6 | To investigate the causal relationship between module gene expression and MN, we conducted a bidirectional Mendelian Randomization (MR) analysis using the TwoSampleMR package (version 0.5.7). eQTL data were obtained from the IEU OpenGWAS database and NephQTL. Outcome data for MN were obtained from the IEU OpenGWAS database, incorporating datasets from diverse populations, including ebi-a-GCST010005 (2150 cases and 5829 controls of European ancestry) and ebi-a-GCST010004 (1632 cases and 3209 controls of East Asian ancestry) for discovery and validation. |
|  | b) | Participants: Give the eligibility criteria, and the sources and methods of selection of participants. Report the sample size, and whether any power or sample size calculations were carried out prior to the main analysis | 6 | Outcome data for MN were obtained from the IEU OpenGWAS database, incorporating datasets from diverse populations, including ebi-a-GCST010005 (2150 cases and 5829 controls of European ancestry) and ebi-a-GCST010004 (1632 cases and 3209 controls of East Asian ancestry) for discovery and validation. |
|  | c) | Describe measurement, quality control and selection of genetic variants | 6 | We performed clumping to select suitable instrumental variables, excluding SNPs in linkage disequilibrium (r²>0.001) or lacking genome-wide significance (p≥5×10⁻⁸). Instrument strength was assessed using the Fstatistic, with F>10 indicating robustness. |
|  | d) | For each exposure, outcome, and other relevant variables, describe methods of assessment and diagnostic criteria for diseases | NA | We used GWAS summary data from a public dataset. It can be obtained from the corresponding references. |
|  | e) | Provide details of ethics committee approval and participant informed consent, if relevant | NA | Not relevant. |
| 5 | **Assumptions** | Explicitly state the three core IV assumptions for the main analysis (relevance, independence and exclusion restriction) as well assumptions for any additional or sensitivity analysis | 7 | We used the inverse-variance weighted (IVW) method for causal assessment, conducted heterogeneity testing to confirm IVW applicability, and assessed pleiotropy with the MR-Egger intercept test. |
| 6 | **Statistical methods: main analysis** | Describe statistical methods and statistics used |  |  |
|  | a) | Describe how quantitative variables were handled in the analyses (i.e., scale, units, model) | NA | We used GWAS summary data from a public dataset. It can be obtained from the corresponding references. |
|  | b) | Describe how genetic variants were handled in the analyses and, if applicable, how their weights were selected | NA | We used GWAS summary data from a public dataset. It can be obtained from the corresponding references. |
|  | c) | Describe the MR estimator (e.g. two-stage least squares, Wald ratio) and related statistics. Detail the included covariates and, in case of two-sample MR, whether the same covariate set was used for adjustment in the two samples | 7 | We used the inverse-variance weighted (IVW) method for causal assessment, conducted heterogeneity testing to confirm IVW applicability, and assessed pleiotropy with the MR-Egger intercept test. |
|  | d) | Explain how missing data were addressed | NA | We used GWAS summary data from a public dataset. It can be obtained from the corresponding references. |
|  | e) | If applicable, indicate how multiple testing was addressed | NA | Due to exploratory findings, we did not perform multiple test correction, and the MR Results will be further verified by ELISA. |
| 7 | **Assessment of assumptions** | Describe any methods or prior knowledge used to assess the assumptions or justify their validity | NA | We suggest possible correlation hypotheses from the previous hdWGCNA analysis |
| 8 | **Sensitivity analyses and additional analyses** | Describe any sensitivity analyses or additional analyses performed (e.g. comparison of effect estimates from different approaches, independent replication, bias analytic techniques, validation of instruments, simulations) | 6 | Outcome data for MN were obtained from the IEU OpenGWAS database, incorporating datasets from diverse populations, including ebi-a-GCST010005 (2150 cases and 5829 controls of European ancestry) and ebi-a-GCST010004 (1632 cases and 3209 controls of East Asian ancestry) for discovery and validation. We used the inverse-variance weighted (IVW) method for causal assessment, conducted heterogeneity testing to confirm IVW applicability, and assessed pleiotropy with the MR-Egger intercept test. Finally, reverse MR analysis was performed to explore reverse causality between gene expression and MN. |
| 9 | **Software and pre-registration** |  |  |  |
|  | a) | Name statistical software and package(s), including version and settings used | 6 | To investigate the causal relationship between module gene expression and MN, we conducted a bidirectional Mendelian Randomization (MR) analysis using the TwoSampleMR package (version 0.5.7). eQTL data were obtained from the IEU OpenGWAS database and NephQTL[25]. |
|  | b) | State whether the study protocol and details were pre-registered (as well as when and where) | NA | Have not pre-registered. |
|  | **RESULTS** |  |  |  |
| 10 | **Descriptive data** |  |  |  |
|  | a) | Report the numbers of individuals at each stage of included studies and reasons for exclusion. Consider use of a flow diagram | NA | We used GWAS summary data from a public dataset. The number of samples for each dataset is described in the M&M. Others can be obtained from the corresponding references. |
|  | b) | Report summary statistics for phenotypic exposure(s), outcome(s), and other relevant variables (e.g. means, SDs, proportions) | NA | We used GWAS summary data from a public dataset. It can be obtained from the corresponding references. |
|  | c) | If the data sources include meta-analyses of previous studies, provide the assessments of heterogeneity across these studies | NA | The data sources do not include meta-analyses of previous studies |
|  | d) | For two-sample MR:  i.  Provide justification of the similarity of the genetic variant-exposure associations between the exposure and outcome samples  ii.  Provide information on the number of individuals who overlap between the exposure and outcome studies | 12 | i To determine the causal relationship between key module gene expression and MN occurrence, bidirectional Mendelian randomization (MR) was conducted using eQTL data and GWAS summary data from two distinct MN cohorts with varying pedigrees. In the European ancestry discovery cohort, potential associations with MN were identified for 17 genes (Figure 7A). The directional trend of OR values for these candidate genes was observed to persist in the East Asian pedigree validation cohort.  ii There is no overlap between the exposure and outcome studies. |
| 11 | **Main results** |  |  |  |
|  | a) | Report the associations between genetic variant and exposure, and between genetic variant and outcome, preferably on an interpretable scale | NA | eQTL correlated with gene expression levels. |
|  | b) | Report MR estimates of the relationship between exposure and outcome, and the measures of uncertainty from the MR analysis, on an interpretable scale, such as odds ratio or relative risk per SD difference | 12 | In the European ancestry discovery cohort, potential associations with MN were identified for 17 genes (Figure 7A). The directional trend of OR values for these candidate genes was observed to persist in the East Asian pedigree validation cohort. However, only SLC1A5 (OR=1.41, p=0.02 in the discovery cohort; OR=1.36, p=0.02 in the validation cohort) and TRIM4 (OR=1.21, p=0.02 in the discovery cohort; OR=1.15, p=0.04 in the validation cohort) maintained statistical significance |
|  | c) | If relevant, consider translating estimates of relative risk into absolute risk for a meaningful time period | NA | No relevance. |
|  | d) | Consider plots to visualize results (e.g. forest plot, scatterplot of associations between genetic variants and outcome versus between genetic variants and exposure) | Figure7A,7B | Figure 7 Bidirectional mendelian randomization unveiling potential causal genes of MN in key modules. (A, B) Forest plot illustrating results of mendelian randomization in the discovery and validation cohort. |
| 12 | **Assessment of assumptions** |  |  |  |
|  | a) | Report the assessment of the validity of the assumptions | 12 | This supported the use of the IVW approach and suggested a direct causal association between the exposure and outcome. In both cohorts, reverse MR analysis confirmed that gene expression levels caused MN and ruled out reverse causation (Figure S3). |
|  | b) | Report any additional statistics (e.g., assessments of heterogeneity across genetic variants, such as *I^2^*, Q statistic or E-value) | 12 | The evaluation of heterogeneity and pleiotropy for the two genes, using Q-value and MR-Egger intercept, showed no significance (p>0.05). |
| 13 | **Sensitivity analyses and additional analyses** |  |  |  |
|  | a) | Report any sensitivity analyses to assess the robustness of the main results to violations of the assumptions | 12 | The evaluation of heterogeneity and pleiotropy for the two genes, using Q-value and MR-Egger intercept, showed no significance (p>0.05). |
|  | b) | Report results from other sensitivity analyses or additional analyses | NA | We do not perform any other sensitivity analyses |
|  | c) | Report any assessment of direction of causal relationship (e.g., bidirectional MR) | 12 | In both cohorts, reverse MR analysis confirmed that gene expression levels caused MN and ruled out reverse causation (Figure S3). |
|  | d) | When relevant, report and compare with estimates from non-MR analyses | NA | In this study, our follow-up experiments demonstrated that SLC1A5 was highly expressed in the serum of MN patients |
|  | e) | Consider additional plots to visualize results (e.g., leave-one-out analyses) | NA | We do not perform such visualization. |
|  | **DISCUSSION** |  |  |  |
| 14 | **Key results** | Summarize key results with reference to study objectives | 13 | Subsequently, a Mendelian randomization approach was employed to confirm the causal relationship between genes within these molecular sets and the onset of MN, using datasets from European and East Asian populations. |
| 15 | **Limitations** | Discuss limitations of the study, taking into account the validity of the IV assumptions, other sources of potential bias, and imprecision. Discuss both direction and magnitude of any potential bias and any efforts to address them | 16 | Additionally, while eQTL data from blood and kidney tissues were included, the absence of single-cell level eQTL data from kidney tissues limits the accuracy of the Mendelian randomization analysis. |
| 16 | **Interpretation** |  |  |  |
|  | a) | Meaning: Give a cautious overall interpretation of results in the context of their limitations and in comparison with other studies | 14 | The study ultimately identified SLC1A5, derived from endothelial cells, as a key gene in MN progression. Validation through 81 serum samples demonstrated that elevated expression of the SLC1A5 protein in MN patients’ serum significantly correlated with urinary protein levels, suggesting that SLC1A5 could serve as a potential diagnostic biomarker and therapeutic target for MN. |
|  | b) | Mechanism: Discuss underlying biological mechanisms that could drive a potential causal relationship between the investigated exposure and the outcome, and whether the gene-environment equivalence assumption is reasonable. Use causal language carefully, clarifying that IV estimates may provide causal effects only under certain assumptions | 15 | In conclusion, SLC1A5-mediated ferroptosis in endothelial cells could result in cytokine release and morphological changes that disrupt the filtration barrier, potentially exposing podocyte antigens and facilitating autoantibody deposition, ultimately triggering MN. |
|  | c) | Clinical relevance: Discuss whether the results have clinical or public policy relevance, and to what extent they inform effect sizes of possible interventions | 15 | Furthermore, elevated serum SLC1A5 protein expression in MN patients correlates significantly with urinary protein levels, indicating its potential as a diagnostic biomarker and monitor for disease progression. |
| 17 | **Generalizability** | Discuss the generalizability of the study results (a) to other populations, (b) across other exposure periods/timings, and (c) across other levels of exposure | NA | We found this association in two different ethnic cohorts. |
|  | **OTHER INFORMATION** |  |  |  |
| 18 | **Funding** | Describe sources of funding and the role of funders in the present study and, if applicable, sources of funding for the databases and original study or studies on which the present study is based | 16 | This work was supported by Science Foundation of Jiangsu Province Grant BK20240371 and BK2023119; Science and Technology Project of Suzhou SKY2022141; National Science Foundation of China Grants 81700589 and 81901632. |
| 19 | **Data and data sharing** | Provide the data used to perform all analyses or report where and how the data can be accessed, and reference these sources in the article. Provide the statistical code needed to reproduce the results in the article, or report whether the code is publicly accessible and if so, where | NA | To investigate the causal relationship between module gene expression and MN, we conducted a bidirectional Mendelian Randomization (MR) analysis using the TwoSampleMR package (version 0.5.7). eQTL data were obtained from the IEU OpenGWAS database and NephQTL. Outcome data for MN were obtained from the IEU OpenGWAS database, incorporating datasets from diverse populations, including ebi-a-GCST010005 (2150 cases and 5829 controls of European ancestry) and ebi-a-GCST010004 (1632 cases and 3209 controls of East Asian ancestry) for discovery and validation. The code is provided in submission system. |
| 20 | **Conflicts of Interest** | All authors should declare all potential conflicts of interest | 16 | The authors declare that they have no conflicts of interest with the contents of this article. |

This checklist is copyrighted by the Equator Network under the Creative Commons Attribution 3.0 Unported (CC BY 3.0) license.

1. Skrivankova VW, Richmond RC, Woolf BAR, Yarmolinsky J, Davies NM, Swanson SA, et al. Strengthening the Reporting of Observational Studies in Epidemiology using Mendelian Randomization (STROBE-MR) Statement. JAMA. 2021;under review.

2. Skrivankova VW, Richmond RC, Woolf BAR, Davies NM, Swanson SA, VanderWeele TJ, et al. Strengthening the Reporting of Observational Studies in Epidemiology using Mendelian Randomisation (STROBE-MR): Explanation and Elaboration. BMJ. 2021;375:n2233.
