# Supplementary material for: Elevated SLC1A5 links to inflamed endothelial cells and proteinuria in membranous nephropathy patients
Source: PeerJ. 2025 Oct 31;13:e20271. doi: 10.7717/peerj.20271 (PMC12581921; doi:10.7717/peerj.20271)

Supplementary Figure Legends

Figure S1 Batch correction and pseudotime starting point estimation in scRNA seq.

(A, B) UMAP visualization colored by sample source (A) and disease state (B). (C) Relative expression of differentially expressed genes identified in bulk transcriptome across each single-cell clusters. (D, E) Macrophage differentiation order identified by Cytotrace. (F, G) Endothelial cell differentiation order identified by Cytotrace. MN, membranous nephropathy; NOM, normal kidney; PT, proximal tubular cells; MC, mesangial cells; Pod, podocytes; LOH, Loop of Henle cells; DT, distal tubular cells; IC, intercalated cells; PC, principal cells; Endo, endothelial cells; Fib/Per, fibroblasts/pericytes; Less diff., less differentiated; More diff, more differentiated.

Figure S1

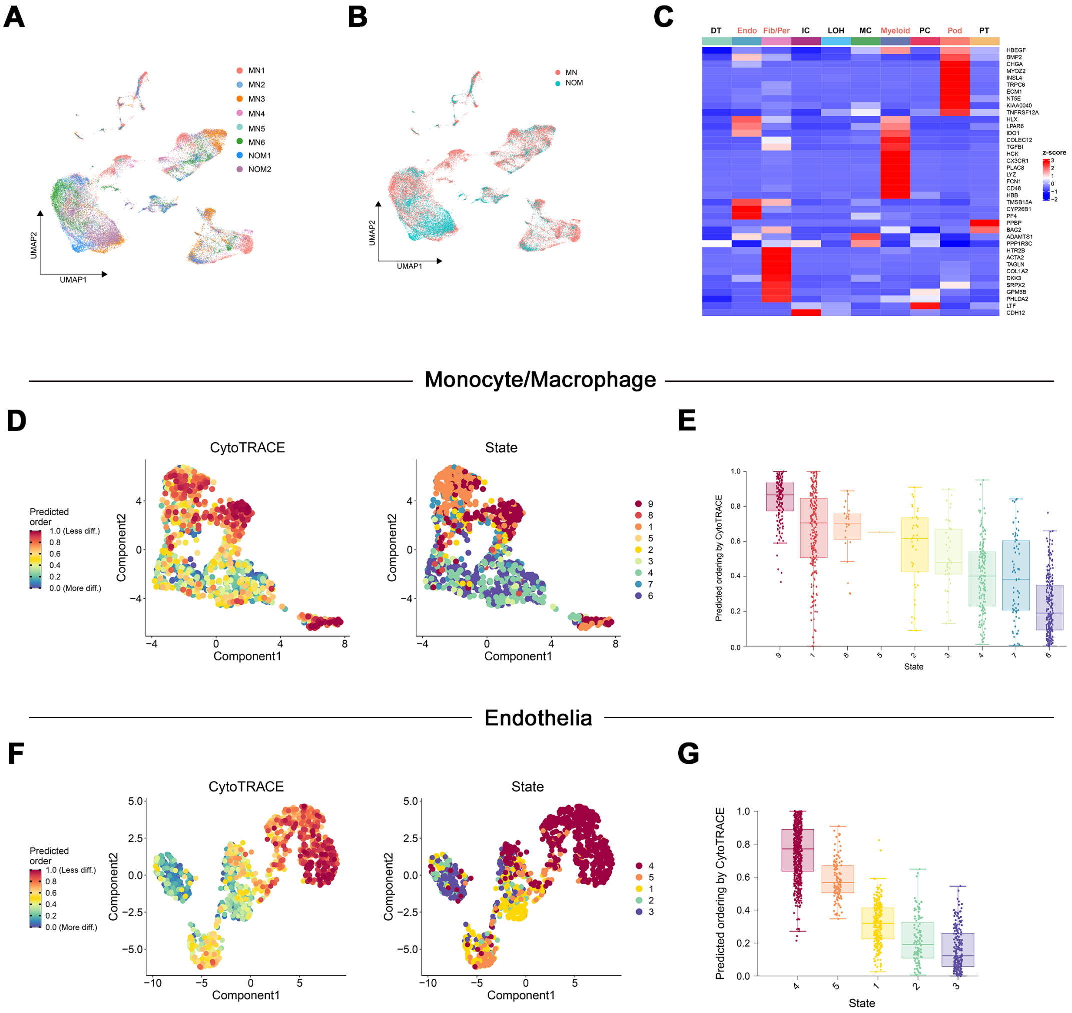
A
MN1
MN2
MN3
MN4
MN5
MN6
NOM1
NOM2
UMAP2
UMAP1
B
MN
NOM
UMAP2
UMAP1
C
DT
Endo
Fib/Per
IC
LOH
MC
Myeloid
PC
Pod
PT
HBEGF
BMP2
CHGA
MYOZ2
INSL4
TRPC6
ECM1
NT5E
KIAA0040
TNFRSF12A
HLX
LPAR6
IDO1
COLEC12
TGFBI
HCK
CX3CR1
PLAC8
LYZ
FCN1
CD48
HBB
TMSB15A
CYP26B1
PF4
PPBP
BAG2
ADAMTS1
PPP1R3C
HTR2B
ACTA2
TAGLN
COL1A2
DKK3
SRPX2
GPM6B
PHLDA2
LTF
CDH12
z-score
Monocyte/Macrophage
D
CytoTRACE
State
Predicted order
1.0 (Less diff.)
0.0 (More diff.)
Component2
Component1
E
Predicted ordering by CytoTRACE
State
Endothelia
F
CytoTRACE
State
Predicted order
1.0 (Less diff.)
0.0 (More diff.)
Component2
Component1
G
Predicted ordering by CytoTRACE
State

Figure S2 Relative expression of differentially expressed genes identified in Olink proteomics across each single-cell clusters.

Figure S2

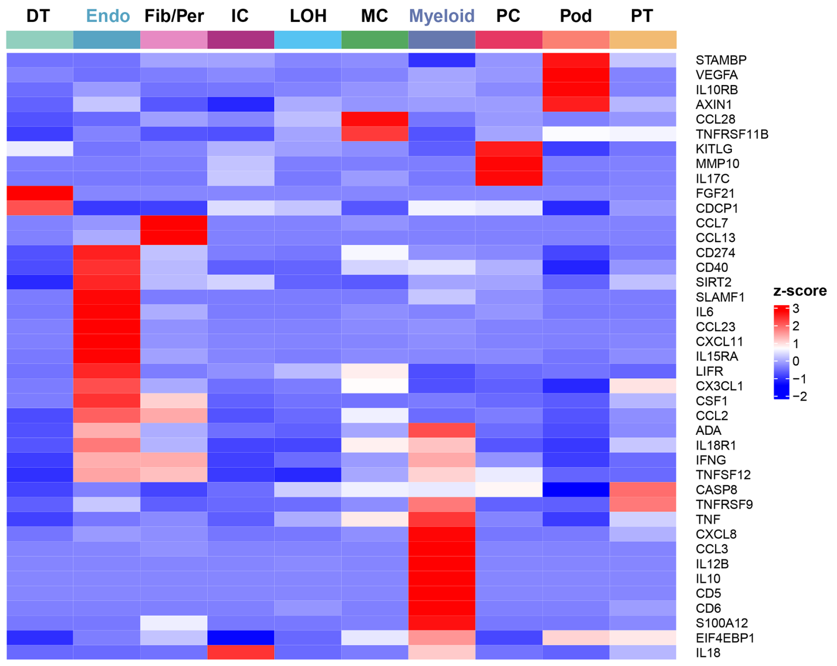

Figure S3 Results of reverse mendelian randomization in the discovery and validation cohort.

Figure S3

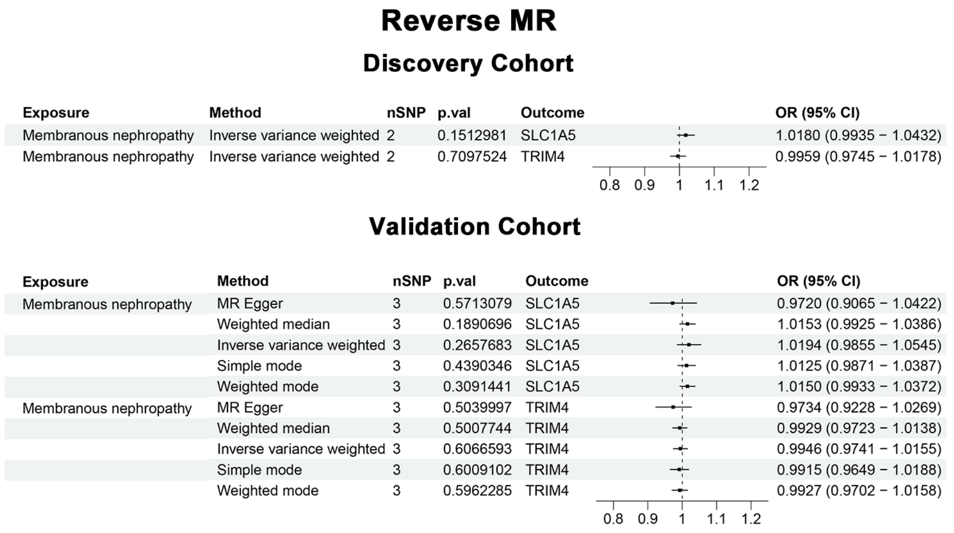

Figure S4 UMAP visualization of *TRIM4* expression in endothelial cells.

Figure S4

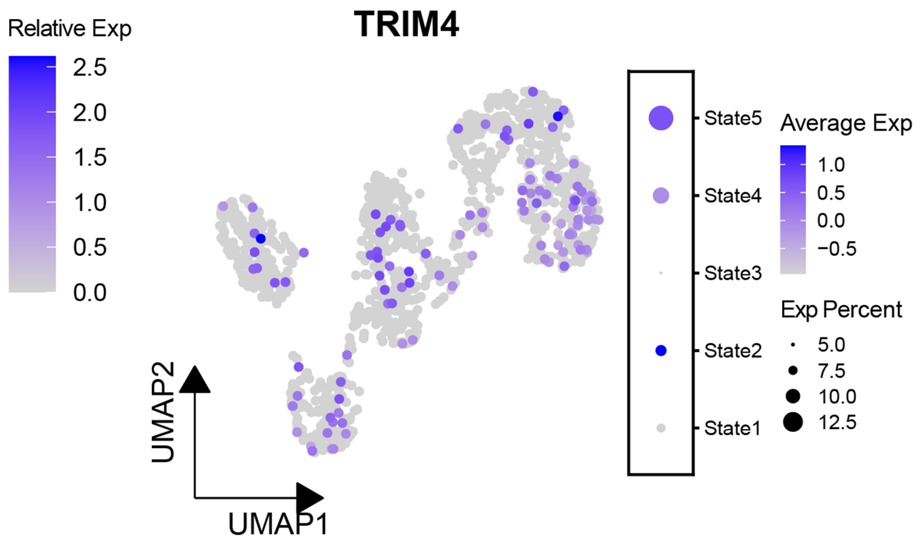

Supplement: Supplemental Information 7 [file peerj-13-20271-s007.pdf]
