# Supplementary material for: Elevated SLC1A5 links to inflamed endothelial cells and proteinuria in membranous nephropathy patients
Source: PeerJ. 2025 Oct 31;13:e20271. doi: 10.7717/peerj.20271 (PMC12581921; doi:10.7717/peerj.20271)
Supplement: Supplemental Information 9 [file peerj-13-20271-s009.docx]

Table S2. The basic characteristics of MN patients and healthy controls

| Variables | Median of MN (P25, P75) | Median of healthy control (P25, P75) | P value |
| --- | --- | --- | --- |
| Gender (M/F) | 31/19 | 15/16 | 0.256 |
| Age (year) | 46.00(36.25-59.50) | 42.00(36.00-59.00) | 0.403 |
| SLC1A5 (pg/ml) | 135.72(122.09-143.14) | 22.50(20.00 -26.00) | <0.001 |
| 24h UPRO (g/d) | 4.31(2.59-5.84) |  |  |
